# Update on the prevalence of allergic sensitization to Russian thistle in South-eastern Ontario: retrospective chart review

**DOI:** 10.1186/1710-1492-7-S2-A19

**Published:** 2011-11-14

**Authors:** Nina Lakhani, Anne K Ellis

**Affiliations:** 1 Department of Medicine, Queen’s University, Kingston, ON, Canada; 2 Department of Biomedical and Molecular Sciences, Queen’s University, Kingston, ON Canada

## Background

Russian thistle (RT) was identified as a potentially clinically significant allergen in phase three of the NHANES survey with over 15% of tested individuals having positive skin tests. Previously estimated prevalence rates of RT skin positivity in Kingston and surrounding catchment area were ~10%. RT was subsequently added to a standard allergen skin testing panel at Queen’s University’s Allergy clinic. 

## Objective

To determine the updated prevalence rate of skin test positivity to Russian Thistle in patients from Kingston and the Southeastern Ontario area, in an unselected patient population.

## Methods

A retrospective chart review documented the rate of sensitization to RT extract (ALK-Abello).  Only patients with appropriate histamine responses were included. Demographic data, presence of relevant clinical symptoms and skin test responses to RT and other cross-reacting allergens were recorded.

## Results

609 charts were reviewed and 304 patients underwent skin testing for RT. Of these, 43 (13.8%) were positive. Of the test-positive cohort, 86% (37/43) had concomitant symptoms of allergic rhinitis/asthma. 41% (18/43) had symptoms that correlated with the predominant RT pollen season. 93% and 58% of these persons had concomitant positive skin tests to ragweed and birch; allergens with known cross-reactivity. 

## Conclusions

This suggests the prevalence of skin test positivity to Russian thistle in Kingston and surrounding area to be approximately 14%, with over 40% of patients reporting correlating symptoms. A higher degree of cross-reactivity with ragweed than previously known may exist. Continuing to include Russian thistle as part of routine allergen testing may further establish its clinical significance.

